# Evaluation of Systemic Microcirculatory Vessel Density in the Early Postoperative Period of Heart Valve Surgery: an Observational Study

**DOI:** 10.21470/1678-9741-2024-0039

**Published:** 2025-06-11

**Authors:** Marcos Vinícius Fernandes, Andrea de Lorenzo, Eduardo Tibiriça

**Affiliations:** 1 Instituto Nacional de Cardiologia, Rio de Janeiro, Rio de Janeiro, Brazil

**Keywords:** Capillares, Milronone, Reference Standards, Microcirculation, Cardiopulmonary Bypass, Microvessels, Point-of-Care-Systems

## Abstract

**Introduction:**

The present study evaluated systemic microcirculatory alterations occurring in the early postoperative period of cardiopulmonary bypass-assisted heart valve surgery compared to preoperative parameters through noninvasive point-of-care microcirculatory imaging of the sublingual area using incident dark field imaging.

**Methods:**

This was a single-center cross-sectional observational study that included 23 patients aged 49 ± 13 years. Sublingual microcirculatory density and perfusion were evaluated using a handheld camera based on incident dark field imaging before surgery and in the early postoperative period.

**Results:**

The total number of capillary vessels (1029 ± 13, P=0.0006), total length of capillary vessels (29.4 ± 3.2 mm, P=0.0005), and capillary vessel density (16.8 ± 1.8 mm/mm2, P=0.0005) were all higher after surgery. On the other hand, the total number of noncapillary vessels (85 ± 34, P=0.05), total length of noncapillary vessels (1.9 ± 0.8 mm, P=0.07), and noncapillary vessel density (1.1 ± 0.5 mm/mm^2^, P=0.07) were similar before and after surgery. The total number of capillary vessels was higher after surgery (1109 ± 92) in patients who received milrinone infusion (P=0.002) but not in patients who did not receive milrinone (986 ± 129, P=0.05).

**Conclusion:**

After cardiac valve surgery, there was an improvement in microvascular parameters concerning capillary vessels and in the total number of microvessels. Moreover, significant positive correlations were found between the use of milrinone and these parameters. The study demonstrated the usefulness of handheld cameras for bedside evaluation of the microcirculation.

## INTRODUCTION

**Table:** 

Abbreviations, Acronyms & Symbols				
CPB	= Cardiopulmonary bypass		MIL	= Milrinone
CVD	= Capillary vessel density		N/A	= Not applicable
DAP	= Diastolic arterial pressure		NCVD	= Noncapillary vessel density
EuroSCORE	= European System for Cardiac Operative Risk Evaluation		NYHA	= New York Heart Association
HVMs	= Handheld vital microscopes		SAP	= Systolic arterial pressure
ICU	= Intensive care unit		SNP	= Sodium nitroprusside
IDF	= Incident dark field		TLCV	= Total length of capillary vessels
IRB	= Institutional Review Board		TLNCV	= Total length of noncapillary vessels
LDPM	= Laser Doppler perfusion monitoring		TNCV	= Total number of capillary vessels
MAP	= Mean arterial pressure		TNNCV	= Total number of noncapillary vessels
MFI	= Microvascular flow index		TVN	= Total vessel number

Surgical procedures are important options for the treatment of heart valve disease^[1]^. In the spectrum of valvular surgery, interventions for degenerative valve disease are predominant in highly developed countries, while the rheumatic etiology is still frequent in many parts of the world^[1^,^2]^.

Cardiac surgeries pose significant physiological stress for a variety of reasons, including cardiopulmonary bypass (CPB). CPB is an essential practice for most cardiac surgical procedures; nevertheless, it induces a complex systemic inflammatory response and coagulation system activation, and subsequent organ dysfunction can result in various postoperative complications^[3]^. The role of systemic microcirculatory dysfunction in this response is well recognized, emphasizing the importance of adequate microcirculatory blood flow to ensure appropriate organ perfusion and oxygen delivery to tissues^[4^,^5]^. In this context, we have already shown that systemic microvascular function evaluated at the skin of the forehead - using laser Doppler perfusion monitoring (LDPM) coupled with thermal hyperemia - is mostly maintained at normothermic circulatory arrest during CPB in patients undergoing coronary artery bypass grafting^[6]^. Using LDPM, we also showed that systemic endothelium-dependent microvascular reactivity is transiently impaired during CPB in children during surgery for the correction of cyanotic and acyanotic congenital heart disease^[7]^. Moreover, this microvascular alteration appears to be related to a reduced systemic bioavailability of nitric oxide, resulting from the inflammatory and pro-oxidative response typical of this surgical setup^[8]^.

The clinical introduction of handheld vital microscopes (HVMs) made it feasible to monitor systemic microcirculation using the vascular bed of the sublingual area in real time at the bedside^[5^,^9^,^10]^. Microcirculation is the primary site of oxygen and nutrient exchange and is essential for the maintenance of vital organ function. It is noteworthy that microcirculatory alterations can occur even when global systemic hemodynamics are preserved, resulting in functional decoupling of the macrocirculation and microcirculation, a phenomenon also known as “hemodynamic incoherence”^[9^-^11]^. The loss of hemodynamic coherence occurs when the correction of systemic hemodynamic variables is not effective in improving microcirculatory perfusion and oxygen delivery to the tissues in order to preserve organ function^[9]^. Accordingly, microcirculatory derangement despite adequate macrocirculation parameters has been correlated with organ dysfunction and reduced survival in different clinical conditions^[12^-^15]^, including the early postoperative period (24 hours) of CPB-assisted cardiac surgery^[16]^. In the context of surgical procedures, a systematic review and meta-analysis designed to investigate the presence of sublingual microcirculatory flow alterations during the immediate and early postoperative period showed that the perfused vessel density and microvascular flow index (MFI) decreased postoperatively^[17]^.

In view of these findings, and despite the paucity of studies on the use of HVMs in cardiac surgeries, the method appears to be useful for real-time, noninvasive, bedside postoperative monitoring, offering complementary information when compared to clinical evaluation and macrocirculatory monitoring^[5^,^18]^. Therefore, the present study sought to evaluate the systemic microcirculatory alterations occurring in the early postoperative period of CPB-assisted heart valve surgery compared to preoperative parameters with noninvasive point-of-care microcirculatory imaging of the sublingual area using incident dark field (IDF) imaging^[5^,^19]^.

## METHODS

The present study was conducted in accordance with the Helsinki Declaration, revised in 2013, and was approved by the Institutional Review Board (IRB) of the Instituto Nacional de Cardiologia (Rio de Janeiro, Brazil), under protocol #CAAE 60999822.3.0000.5272 and registered at ClinicalTrials.gov under protocol #NCT05728047. Once deemed eligible for participation in this study, all subjects read and signed an informed consent form approved by the IRB.

The reporting of this study follows the recommendations of the Strengthening the Reporting of Observational Studies in Epidemiology (or STROBE) statement^[20]^.

### Study Design

This was a single-center cross-sectional observational study that included 23 patients aged 49 ± 13 years who were consecutively enrolled during a period of four months and scheduled for heart valve surgery with CPB at a quaternary public hospital, namely, the Instituto Nacional de Cardiologia, Ministry of Health, Rio de Janeiro, Brazil. Study exclusion criteria included age < 18 years, combined heart surgeries, and infective endocarditis. Once deemed eligible for participation in this study, all subjects read and signed an informed consent form approved by the IRB.

### Evaluation of Systemic Microvascular Parameters

Sublingual microcirculatory density and perfusion were assessed using a handheld camera based on IDF imaging (Cytocam, Braedius Medical, Huizen, The Netherlands), as previously described^[18]^. It has been recently demonstrated that the sublingual region has a homogenous spatial distribution of most microvascular parameters, including total and functional vessel density^[21]^.

Each recruited patient was evaluated in two stages: 1) during hospitalization, in the preoperative period of cardiac surgeries to be performed within the next 48 hours; and 2) in the early postoperative phase of cardiac surgery, within the first four hours of the patient's arrival at the intensive care unit, while still under residual sedation and orotracheal intubation and mechanical ventilation.

At each moment, the microscope was gently positioned under the patient's tongue until we obtained adequate visualization of the sublingual microcirculation after focus and contrast adjustment. At least three videos of five seconds each were obtained, with attention given to their quality and particularly to the proper location and the absence of pressure artifacts and excess saliva.

### Microcirculatory Image Analysis

Offline image analysis was performed in patients who had recordings with good or acceptable image quality according to the second consensus on the assessment of sublingual microcirculation of the European Society of Intensive Care Medicine^[22]^, using CytoCamTools 3.1.4 software (Braedius Medical, Huizen, The Netherlands).

Analysis of capillary vessels in the images (diameter range between 6.04 and 15.9 µm) included the capillary diameter (µm), total number of capillary vessels (TNCV), total length of capillary vessels (TLCV) (mm), and capillary vessel density (CVD) (mm/mm^2^).

Analysis of noncapillary vessels (diameter 16-50 µm) in the images included the noncapillary vessel diameter (µm), total number of noncapillary vessels (TNNCV), total length of noncapillary vessels (TLNCV) (mm), and noncapillary vessel density (NCVD) (mm/mm2). The total vessel number (TVN) represents the total number of vessels with diameters < 50 µm.

Finally, videos were analyzed in a blinded fashion for calculation of the MFI, as previously described^[22]^. MFI is a semiquantitative score that distinguishes between no flow (0), intermittent flow (1), sluggish flow (2), and continuous flow (3). A score was assigned to each quadrant of the video screen. Scores of the four quadrants were averaged per video, and values from three videos were averaged.

### Statistical Analysis

The prospective analysis of statistical power was based on data from previous studies from Ince et al.^[23^,^24]^, using the technique of evaluation of the sublingual microcirculation with IDF-based Cytocam. The study showed an increase in microcirculatory density from 10.5 ± 1.2 to 12.9 ± 1.2 mm/mm^2^ following red blood cell transfusion in patients undergoing on-pump cardiac surgery^[24]^. A power of 95% and an alpha of 0.05 were used in the calculations and indicated that the minimum sample size was eight patients.

The distribution of values was analyzed using the Shapiro-Wilk normality test. The results were analyzed using a two-tailed paired *t*-test or paired Wilcoxon test (Wilcoxon signed-ranks test) for values with parametric and nonparametric distributions, respectively. *P*-values < 0.05 were considered significant. Statistical analyses were performed using Prism version 7.0 software (GraphPad Software, La Jolla, California, United States of America). Numerical data were correlated using Pearson's correlation test or Spearman's correlation test according to the necessary assumptions. The linear regression model was developed for multivariate data analysis. The selection of variables for the composition of the multivariate model was performed with the stepwise technique using the criterion of statistical significance of the variables with the response analyzed in the regression (R software, R Core Team, 2021).

## RESULTS

### Clinical Characteristics of Patients and Surgical Data

Demographic data, patient characteristics, and surgical details are presented in Table 1. During the immediate postoperative period in the intensive care unit, 48% (n=11) of patients were treated with intravenous infusions of noradrenaline, 35% (n=8) with milrinone, 13% (n=3) with sodium nitroprusside, and only one patient with vasopressin (Table 1). The systolic, diastolic, and mean arterial pressures were not different before and after surgery (Table 2).

**Table 1:** Clinical characteristics of the patients and surgical data (n=23).

Clinical parameters	
Age (years)	49 ± 13
Male sex, n (%)	9 (39)
Body weight (kg)	67 ± 18
Body mass index (kg/m^2^)	25.6 (21.6-27.1)
EuroSCORE (%)	3.2 (2.1-4.3)
NYHA	
I, n (%)	2 (9)
II, n (%)	16 (70)
III, n (%)	4 (17)
IV, n (%)	1(4)
Left ventricular ejection fraction (%, Teicholz)	64.4 ± 9.7
Arterial hypertension, n (%)	9 (39)
Diabetes, n (%)	3 (13)
Dyslipidemia, n (%)	4 (17)
Smoking, n (%)	8 (35)
Stroke, n (%)	4 (17)
Rheumatic fever, n (%)	12 (52)
Atrial fibrillation, n (%)	6 (26)
Previous cardiac surgery, n (%)	11 (48)
Surgical parameters	
Types of surgeries	
Aortic valve replacement, n (%)	7 (30)
Aortic valve repair, n (%)	2 (9)
Mitral valve replacement, n (%)	10 (44)
Mitral valve repair, n (%)	3 (13)
Tricuspid valve replacement, n (%)	1 (4)
Types of valves	
Mechanical valve, n (%)	10 (55.6)
Biological tissue valve, n (%)	8 (44.4)
Cardiopulmonary bypass time (min)	146 ± 42
Aortic cross-clamping time (min)	127 ± 41
Perioperative mortality, n (%)	0 (0)
ICU noradrenaline, n (%)	11 (48)
ICU noradrenaline dose (µg/kg/min)	0.1 (0.08-0.35)
ICU vasopressin, n (%)	1 (4)
ICU sodium nitroprusside, n (%)	3 (13)
ICU sodium nitroprusside dose (µg/kg/min)	0.33 ± 0.15
ICU milrinone, n (%)	8 (35)
ICU milrinone dose (µg/kg/min)	0.48 ± 0.12

Data are presented as the mean ± standard deviation or as medians (25^th^ - 75^th^ percentiles) for values that did not fit a Gaussian distribution (by the Shapiro-Wilk normality test)

EuroSCORE=European System for Cardiac Operative Risk Evaluation; ICU=intensive care unit; NYHA=New York Heart Association

**Table 2:** Clinical characteristics of patients before and after surgery (n=23).

Clinical parameters	Before surgery	After surgery	*P*-values
SAP (mmHg)	110 ± 12	109 ± 22	0.94
DAP (mmHg)	69 ± 11	65 ± 15	0.29
MAP (mmHg)	82 ± 10	81 ± 18	0.80
Heart rate (bpm)	72 ± 10	82 ± 12	0.003
Hemoglobin (g/dL)	12 ± 1.4	10.5 ± 1.2	< 0.0001
Hematocrit (%)	39 ± 4.6	31.7 ± 4.2	< 0.0001
Leukocytes (mm^3^)	6,500 (5,700-7,200)	14,700 (13,900-18,300)	< 0.0001
Platelets (mm^3^)	213,304 ± 48,302	168,826 ± 42,566	0.0006
Creatinine (mg/dL)	0.9 ± 0.1	1.1 ± 0.4	0.0015
SaO2 (%)	N/A	99 ± 1	N/A
Plasma pH	N/A	7.37 ± 0.05	N/A
Serum bicarbonate (mEq/L)	N/A	23.3 ± 1.8	N/A
Plasma lactate (mmol/L)	N/A	2.69 ± 1.3	N/A

Data are presented as the mean ± standard deviation or as medians (25^th^ - 75^th^ percentiles) for values that did not fit a Gaussian distribution (by the Shapiro-Wilk normality test)

Values in bold denote statistical significance

DAP=diastolic arterial pressure; MAP=mean arterial pressure; N/A=not applicable; SAP=systolic arterial pressure

### Microcirculatory Parameters

The mean diameters of capillary vessels and noncapillary vessels were 10.96 ± 0.01 and 26.49 ± 1.6 µm, respectively.

#### Capillary Vessels

**Fig. 1:**
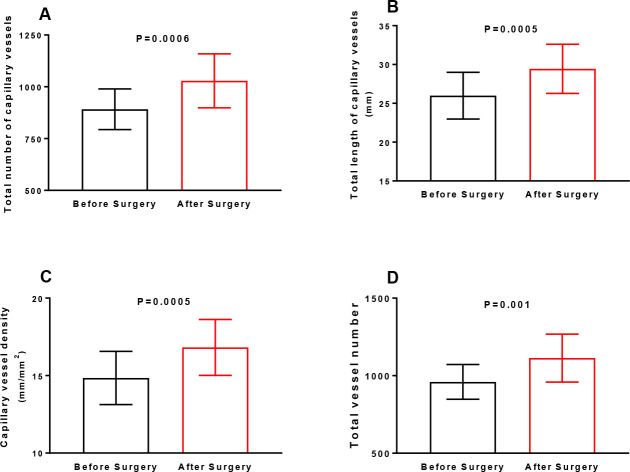
(A) Total number of capillary vessels, (B) total length of capillary vessels, (C) capillary vessel density, and (D) total vessel number in the sublingual area assessed using a handheld camera based on incident dark field imaging before and during the early postoperative period of heart valve surgery (n=23). The results are shown as the means ± standard deviations (Shapiro-Wilk normality test). Statistical analyses were performed using paired Student’s t-tests.

TNCV was higher after surgery (1029 ± 13) than before surgery (891 ± 98; *P*=0.0006) (Figure 1A). TLCV was also higher after surgery (29.4 ± 3.2 mm) than before surgery (25.9 ± 3.0 mm; *P*=0.0005) (Figure 1B). The same profile was observed for CVD: 16.8 ± 1.8 and 14.8 ± 1.7 mm/mm^2^ after and before surgery, respectively (*P*=0.0005) (Figure 1C). TVN was also increased after surgery (1114 ± 154) compared to values before surgery (960 ± 112, *P*=0.001) (Figure 1D).

#### Noncapillary Vessels

**Fig. 2:**
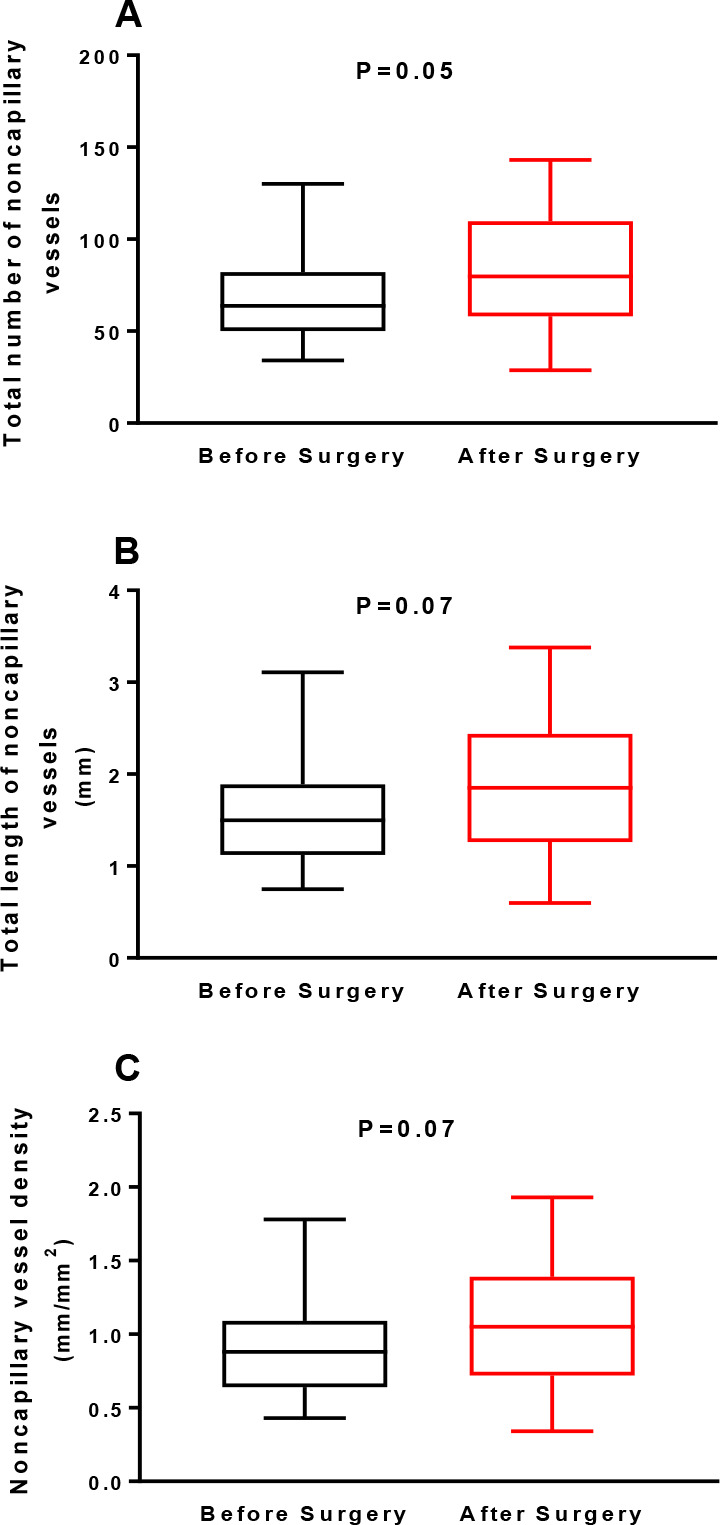
(A) Total number of noncapillary vessels, (B) total length of noncapillary vessels, and (C) noncapillary vessel density in the sublingual area assessed using a handheld camera based on incident dark field imaging before and during the early postoperative period of heart valve surgery (n=23). Values are expressed as box and whisker plots where the center line denotes the median value, the box contains the 25^th^ to 75^th^ percentiles of dataset and whiskers mark the maximum and minimum values (Shapiro-Wilk normality test). Statistical analyses were performed using the Wilcoxon matched-pairs signed rank test.

TNNCV did not differ after (85 ± 34) compared to before surgery (68 ± 24) (*P*=0.05) (Figure 2A). TLNCV was also similar after (1.9 ± 0.8 mm) or before surgery (1.6 ± 0.6 mm) (*P*=0.07) (Figure 2B). Finally, NCVD was also similar after (1.1 ± 0.5 mm/mm^2^) and before surgery (0.9 ± 0.3 mm/mm^2^) (*P*=0.07) (Figure 2C).

### Microvascular Flow Index

**Fig. 3:**
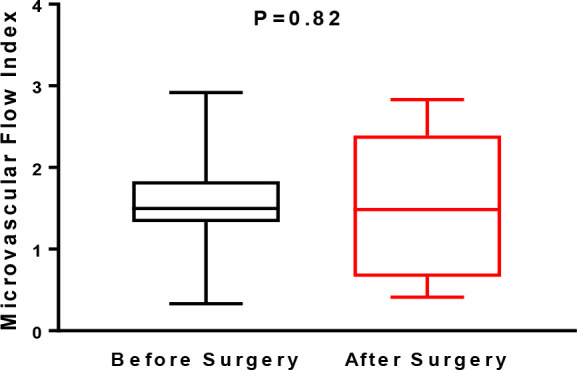
Microvascular flow index in the sublingual area assessed using a handheld camera based on incident dark field imaging before and during the early postoperative period of heart valve surgery (n=23). Values are expressed as box and whisker plots where the center line denotes the median value, the box contains the 25^th^ to 75^th^ percentiles of dataset and whiskers mark the maximum and minimum values (Shapiro-Wilk normality test). Statistical analyses were performed using the Wilcoxon matched-pairs signed rank test.

There were no significant differences between the MFI before (1.50 [1.33-1.83]) and after surgery (1.48 [0.66-2.39]) (*P*=0.82) (Figure 3).

### Microcirculatory Parameters in Patients Receiving or Not Receiving Milrinone Infusion

#### Capillary Vessels

**Fig. 4:**
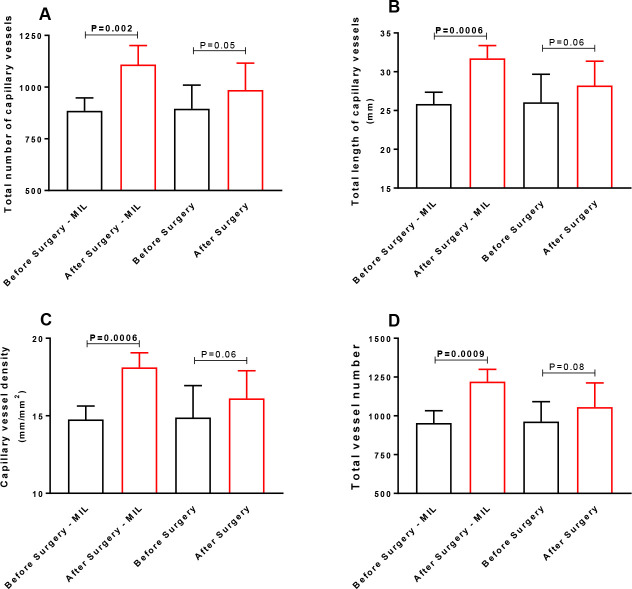
(A) Total number of capillary vessels, (B) total length of capillary vessels, (C) capillary vessel density, and (D) total vessel number in the sublingual area assessed using a handheld camera based on incident dark field imaging before and during the early postoperative period of heart valve surgery in patients receiving (n=8) or not receiving (n=15) milrinone (MIL) continuous infusions after surgery. The results are shown as the means ± standard deviations (Shapiro-Wilk normality test). Statistical analyses were performed using paired Student’s t-tests.

TNCV was higher after surgery in patients who received milrinone infusion during the postoperative period (1109 ± 92) compared with values obtained before surgery (885 ± 62; *P*=0.002) (Figure 4A). Nevertheless, in patients who did not receive milrinone, the TNCV values after surgery were similar (986 ± 129) to the values obtained before surgery (895 ± 114; *P*=0.05) (Figure 4A). The same pattern of response was observed concerning TLCV, which was also higher after surgery (31.7 ± 1.6 mm) than before surgery (25.8 ± 1.5 mm; *P*=0.0006) (Figure 4B) in patients who received milrinone but not in those who did not receive milrinone (after surgery [28.2 ± 3.1 mm] compared to before surgery [26.1 ± 3.6 mm; *P*=0.06] [Figure 4B]). The same profile was observed for CVD (18.1 ± 0.9 and 14.8 ± 0.9 mm/mm^2^ after and before surgery, respectively [*P*=0.0006] [Figure 4C]) in patients who received milrinone and (16.1 ± 1.8 and 14.9 ± 2 mm/mm^2^ after and before surgery, respectively [*P*=0.06] [Figure 4C]) in patients who did not receive milrinone. TVN also increased after surgery (1.221 ± 79) compared to the values before surgery (953 ± 79, *P*=0.0009) (Figure 4D) in patients who received milrinone but not in those who did not receive milrinone (1,056 ± 156 and 963 ± 128 mm/mm^2^ after and before surgery, respectively; *P*=0.08) (Figure 4D).

#### Noncapillary Vessels

**Fig. 5:**
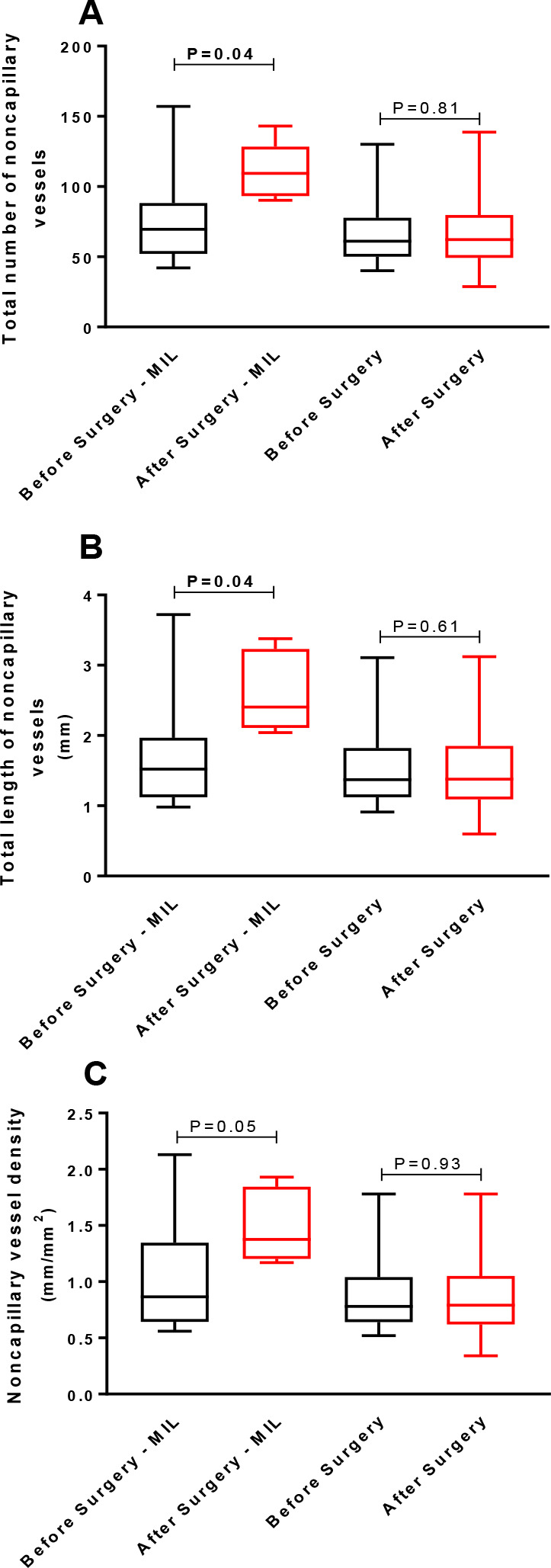
(A) Total number of noncapillary vessels, (B) total length of noncapillary vessels, and (C) noncapillary vessel density in the sublingual area assessed using a handheld camera based on incident dark field imaging before and during the early postoperative period of heart valve surgery in patients receiving (n=8) or not receiving (n=15) milrinone (MIL) continuous infusions after surgery. Values are expressed as box and whisker plots where the center line denotes the median value, the box contains the 25^th^ to 75^th^ percentiles of dataset and whiskers mark the maximum and minimum values (Shapiro-Wilk normality test). Statistical analyses were performed using the Wilcoxon matched-pairs signed rank test.

TNNCV was higher after surgery (112 ± 19) than before surgery (77 ± 36; *P*=0.04) (Figure 5A) in patients who received milrinone but did not differ after (70 ± 31) compared to before surgery (68 ± 26) in patients who did not receive milrinone (*P*=0.81) (Figure 5A). TLNCV was also higher after surgery (2.4 [2.1-3.2] mm) than before surgery (1.5 [1.5-1.9] mm [*P*=0.04] [Figure 5B]) in patients who received milrinone but not in patients who did not receive milrinone (after surgery: 1.4 [1.1-1.8] mm *vs.* before surgery: 1.4 [1.1-1.8] mm [*P*=0.61] [Figure 5B]). Finally, NCVD was similar after surgery (1.5 ± 0.3 mm/mm^2^) and before surgery (1.0 ± 0.5 mm/mm^2^) (*P*=0.05) (Figure 5C) in patients who received milrinone and in those who did not receive milrinone (after surgery: 0.9 ± 0.4 mm/mm^2^ and before surgery: 0.9 ± 0.4 mm/mm^2^; *P*=0.93) (Figure 5C).

### Correlations and Associations Between the Microcirculatory Parameters After Cardiac Surgery and the Clinical or Surgical Parameters

There were significant negative correlations between the CPB time and mean arterial pressure and a positive correlation between the milrinone dose and capillary parameters (Table 3). Noncapillary parameters were positively correlated only with the milrinone dose (Table 3). There were no other correlations of microvascular parameters with clinical or surgical parameters (data not shown). There was also an association of the use of milrinone during the immediate postoperative period in the intensive care unit with higher microvascular density (Table 4).

**Table 3:** Pearson’s or Spearman’s correlations between microvascular parameters after cardiac surgery and clinical or surgical parameters.

	TNCV		TLCV (mm)		CVD (mm/mm^2^)		TVN	
Correlation	r	*P*-value	r	*P*-value	r	*P*-value	r	*P*-value
CPB (min)	-0.463	0.017	-0.447	0.033	-0.446	0.033	-0.463	0.026
Noradrenaline in ICU (dose, µg/kg/min)	0.35	0.10	0.36	0.09	0.36	0.09	0.33	0.12
SNP in ICU (dose, µg/kg/min)	-0.40	0.06	-0.40	0.06	-0.40	0.06	-0.39	0.06
Milrinone in ICU (dose, µg/kg/min)	0.492	0.017	0.557	0.006	0.557	0.006	0.535	0.009
MAP (mmHg)	-0.559	0.006	-0.548	0.007	-0.548	0.007	-0.558	0.006
	TNNCV		TLNCV (mm)		NCVD (mm/mm^2^)			
Correlation	r	*P*-value	r	*P*-value	r	*P*-value		
CPB (min)	-0.331	0.12	-0.317	0.14	-0.318	0.14		
Noradrenaline in ICU (dose, µg/kg/min)	0.179	0.41	0.135	0.54	0.135	0.54		
SNP in ICU (dose, µg/kg/min)	-0.242	0.26	-0.240	0.27	-0.241	0.27		
Milrinone in ICU (dose, µg/kg/min)	0.551	0.006	0.522	0.01	0.522	0.01		
MAP (mmHg)	-0.397	0.06	-0.338	0.11	-0.339	0.11		

Values in bold denote statistical significance

CPB=cardiopulmonary bypass; CVD=capillary vessel density; ICU=intensive care unit; MAP=mean arterial pressure; NCVD=noncapillary vessel density; SNP=sodium nitroprusside; TLCV=total length of capillary vessels; TLNCV=total length of noncapillary vessels; TNCV=total number of capillary vessels; TNNCV=total number of noncapillary vessels; TVN=total vessel number

**Table 4:** Associations between the use of vasopressors or inotropes and microvascular parameters during the immediate postoperative period.

	Milrinone in ICU			Noradrenaline in ICU			SNP in ICU		
Parameter	Yes	No	*P*-value	Yes	No	*P*-value	Yes	No	*P*-value
TNCV	1,108 ± 92	986 ± 129	0.028	1,039 ± 132	1,019 ± 134	0.720	961 ± 262	1,039 ± 107	0.345
TLCV (mm)	31.7 ± 1.6	28.2 ± 3.1	0.008	29.6 ± 3.3	29.2 ± 3.2	0.763	27.5 ± 5.3	29.7 ± 2.8	0.276
CVD (mm/mm^2^)	18.1 ± 0.9	16.1 ± 1.8	0.008	16.9 ± 1.8	16.7 ± 1.8	0.762	15.7 ± 3	16.9 ± 1.6	0.276
TVN	1,220 ± 79	1,056 ± 156	0.011	1,127 ± 155	1,100 ± 159	0.684	1,037 ± 314	1,125 ± 127	0.369
	Milrinone in ICU			Noradrenaline in ICU			SNP in ICU		
Parameter	Yes	No	*P*-value	Yes	No	*P*-value	Yes	No	*P*-value
TNNCV	112 ± 19	70 ± 31	0.002	88 ± 36	82 ± 33	0.630	76 ± 54	86 ± 31	0.641
TLNCV (mm)	2.6 ± 0.5	1.6 ± 0.7	0.002	2 ± 0.9	1.9 ± 0.8	0.764	1.7 ± 1.2	1.9 ± 0.8	0.614
NCVD (mm/mm^2^)	1.5 ± 0.3	0.9 ± 0.4	0.002	1.1 ± 0.5	1.1 ± 0.5	0.762	0.9 ± 0.7	1.1 ± 0.4	0.610

CVD=capillary vessel density; ICU=intensive care unit; NCVD=noncapillary vessel density; SNP=sodium nitroprusside; TLCV=total length of capillary vessels; TLNCV=total length of noncapillary vessels; TNCV=total number of capillary vessels; TNNCV=total number of noncapillary vessels; TVN=total vessel number

Values in bold denote statistical significance

## DISCUSSION

Cardiac surgery is generally a life-saving treatment for many patients, despite being highly invasive and promoting several systemic effects, mostly linked to CPB, including inflammation and endothelial dysfunction[^25^-^27^]. In Brazil, according to the BYPASS registry, rheumatic valve disease is the most frequent indication for surgical valve procedures, followed by congenital etiology, senile degenerative aortic disease, degenerative mitral prolapse or chordae rupture, and infective endocarditis. In that registry, the most frequently performed valvular heart surgery was isolated aortic valve replacement (34% of all valve procedures), followed by mitral valve replacement (25%); the vast majority (98.3%) were conventional, open-chest surgeries, with a very low proportion of minimally invasive surgeries^[28]^.

This study was designed to evaluate patients undergoing valve surgery, as this patient population in Brazil is usually younger, with fewer atherosclerotic risk factors than patients with coronary artery disease, due to the prevalence of the rheumatic etiology^[3]^. Therefore, possible vascular derangements due to atherosclerosis would be minimized, and the observed behavior of the microcirculation might be more directly attributed to the effects of surgery, CPB, or anesthesia.

We observed that, after surgery, patients displayed an increase in all microvascular parameters concerning capillary vessels (TNCV, TLCV, and CVD) and in the total number of microvessels (vessels with diameter < 50 µm); on the other hand, TNNCV and TLNCV, in our cases mostly represented by arterioles (resistance vessels with diameters between 16 and 50 µm)^[29]^, did not increase after surgery in comparison to the preoperative evaluation. The same pattern of response was observed regarding the MFI, with no statistically significant changes. The substantial increase in capillary density observed in the present study - in perioperative patients - points to an improvement in systemic tissue perfusion along with a favorable prognosis with a reduced incidence of perioperative complications, as previously demonstrated^[30^,^31]^.

The observed postoperative responses of capillary vessel parameters might initially be viewed as a result of a global hemodynamic improvement after the correction of the underlying valve disease; however, after searching for possible confounders, significant correlations were found between the use of milrinone and microvascular parameters. Indeed, if patients who received milrinone were excluded from the analysis, only a trend toward improved microcirculatory parameters could be observed. Moreover, in the group of patients receiving milrinone infusion after surgery, the total number and length of both capillary and noncapillary microvessels was positively correlated with the use of milrinone. This effect is probably due to the pharmacological properties of milrinone that distinguish it from other inotropic agents, such as its ability to increase inotropism while generating a significant reduction in peripheral vascular resistance and pulmonary vascular resistance (significant systemic and pulmonary vasodilation)^[32]^.

Milrinone is a phosphodiesterase inhibitor that increases intracellular cyclic adenosine monophosphate, resulting in a positive inotropic effect and peripheral vasodilatation^[33]^. Consequently, it has profound effects on microcirculation, such as the attenuation of capillary perfusion deficits during endotoxemic shock, which is not observed with norepinephrine infusion^[34]^. Milrinone also promotes an increase in cardiac output, which may account for the maintenance of systemic arterial pressure despite widespread vasodilatation. It is important to keep in mind that there is a key difference between cardiogenic and septic shock, which is that in the former, there is also a relationship between cardiac output and microcirculatory status^[35]^, and milrinone has beneficial effects on both. Therefore, a correlation between the use and dose of milrinone and both microand macrovascular parameters was expected. Of note, the absence of significant microcirculatory effects of norepinephrine is similar to previously described data in a sepsis model^[34]^. Although its vasopressor properties are undeniable, norepinephrine does not actually recruit microcirculation but instead reduces microcirculatory perfusion^[36]^. Finally, the absence of correlations between sodium nitroprusside and microcirculatory parameters is similar to prior data showing that it failed to increase the MFI in both sublingual small-sized and large-sized vessels^[37]^.

The association between CPB duration and microcirculatory parameters is also similar to that in prior studies. When comparing on-pump and off-pump cardiac surgery to thyroid surgery, perfused small vessel density was most severely diminished in on-pump cardiac surgery^[38]^.

This study may contribute to the understanding of the behavior of microcirculation after cardiac surgery, a field with relatively less data compared to studies on sepsis^[38^,^39]^. Additionally, it underscores the value of microcirculatory management, as microcirculatory derangements may still exist despite a normal systemic pressure^[17]^. Indeed, although traditional hemodynamic management has focused on macrocirculatory monitoring, microcirculatory dysfunction may go unrecognized but is also related to outcomes^[16]^. With the availability of monitoring tools to evaluate the microcirculation, such as HVMs, clinicians may better understand and manage the microcirculation in surgical patients in the future, with personalized approaches, such as more adequate titration of fluids and vasoactive drugs, which may possibly improve patient prognosis.

### Limitations and Strengths

This was a single-center study from a quaternary-care specialized hospital (cardiovascular surgery), which was designed to test a new methodology of systemic microvascular evaluation in a small number of subjects initially. Thus, it was essentially a hypothesis-generating study, and larger confirmatory studies are needed. Anyhow, this methodology has previously shown to be effective and yield robust results in limited samples of subjects^[23^,^24]^. The IDF video-microscope used in the present study has been shown to provide improved image acquisition of human sublingual microcirculation when compared to earlier models of HVMs^[39]^. Superior in five out of the six categories comprising the microcirculatory image quality scoring system, the use of IDF offers an advanced insight into the clinical evaluation of the microvasculature^[39]^.

Finally, surgical and clinical results might have been influenced by specific practices at the institution, while multi-center studies would be more desirable to assess different settings and patient populations. However, concerning the technique we evaluated, the results are independent of surgical success or specific intraoperative or postoperative practices, as they simply depict the status of the microcirculation, regardless of its improvement or worsening.

## CONCLUSION

After cardiac valve surgery, there was an improvement in microvascular parameters concerning capillary vessels and in the total number of microvessels. These responses might be attributed to hemodynamic improvement after the correction of the underlying valve disease; however, significant correlations were found between the use of milrinone and microvascular parameters, possibly due to increase inotropism with reduction in peripheral vascular resistance and pulmonary vascular resistance promoted by the drug. Nonetheless, the study demonstrated the usefulness of HVMs for bedside evaluation of microcirculation.
